# Robustness analysis of culturing perturbations on *Escherichia coli *colony biofilm beta-lactam and aminoglycoside antibiotic tolerance

**DOI:** 10.1186/1471-2180-10-185

**Published:** 2010-07-07

**Authors:** Trevor R Zuroff, Hans Bernstein, Jenna Lloyd-Randolfi, Lourdes Jimenez-Taracido, Philip S Stewart, Ross P Carlson

**Affiliations:** 1 Department of Chemical and Biological Engineering, Center for Biofilm Engineering, Montana State University, Bozeman MT 59717, USA; 2 Johns Hopkins University, Baltimore Maryland, USA; 3 University of Cadiz, Cadiz, Spain

## Abstract

**Background:**

Biofilms are ubiquitous. For instance, the majority of medical infections are thought to involve biofilms. However even after decades of investigation, the *in vivo *efficacy of many antimicrobial strategies is still debated suggesting there is a need for better understanding of biofilm antimicrobial tolerances. The current study's goal is to characterize the robustness of biofilm antibiotic tolerance to medically and industrially relevant culturing perturbations. By definition, robust systems will return similar, predictable responses when perturbed while non-robust systems will return very different and potentially unpredictable responses. The predictability of an antibiotic tolerance response is essential to developing, testing, and employing antimicrobial strategies.

**Results:**

The antibiotic tolerance of *Escherichia coli *colony biofilms was tested against beta-lactam and aminoglycoside class antibiotics. Control scenario tolerances were compared to tolerances under culturing perturbations including 1) different nutritional environments 2) different temperatures 3) interruption of cellular quorum sensing and 4) different biofilm culture ages. Here, antibiotic tolerance was defined in terms of culturable biofilm cells recovered after a twenty four hour antibiotic treatment.

Colony biofilm antibiotic tolerances were not robust to perturbations. Altering basic culturing parameters like nutritional environment or temperature resulted in very different, non-intuitive antibiotic tolerance responses. Some minor perturbations like increasing the glucose concentration from 0.1 to 1 g/L caused a ten million fold difference in culturable cells over a twenty four hour antibiotic treatment.

**Conclusions:**

The current study presents a basis for robustness analysis of biofilm antibiotic tolerance. Biofilm antibiotic tolerance can vary in unpredictable manners based on modest changes in culturing conditions. Common antimicrobial testing methods, which only consider a single culturing condition, are not desirable since slight culturing variations can lead to very different outcomes. The presented data suggest it is essential to test antimicrobial strategies over a range of culturing perturbations relevant to the targeted application. In addition, the highly dynamic antibiotic tolerance responses observed here may explain why some current antimicrobial strategies occasionally fail.

## Background

Biofilms plague both medical and industrial surfaces and are difficult to treat with common antimicrobial strategies [1,2]. Cells residing within biofilms are often tolerant to antimicrobial agents at concentrations thousands of times higher than what is necessary to eradicate the same cells growing planktonicly (*e.g. *[3,4]). This recalcitrance is likely due to a combination of physical and physiological factors. Cells from a disrupted biofilm typically become susceptible to antibiotics when regrown planktonicly [5-7].

The ubiquity of biofilms and their associated financial costs have inspired intensive antifouling efforts. A widely used anti-biofilm approach is to impregnate surfaces with antiseptics or antibiotics (reviewed in [8,9]). The benefit of antimicrobial impregnated medical devices is still controversial despite decades of research and investment. For example, after reviewing years of studies, McConnell *et al*. [10,11] conclude that more rigorous investigations are required to either support or refute the hypothesis that central venous catheters coated with antimicrobial agents reduce the rate of blood stream infections. While other researchers disagree with these conclusions (*e.g*. [12]), the fact there is still a debate regarding the efficacy of these strategies suggests there is need for better technologies and a better understanding of what parameters influence bacterial tolerance to antimicrobial agents.

The current study aims to characterize colony biofilm antibiotic tolerance as a function of culturing conditions. The colony biofilm model is a widely adopted culturing system which possesses most features included in the numerous attempts to define a biofilm including: high cell density, extracellular polymeric substance, chemical gradients, spatially dependent microbial activities including slow growth, and reduced susceptibility to antibiotics (*e.g*. [4,13-16]). This study utilizes an engineering approach, known as robustness analysis, which is used to analyze complex systems. Robustness analysis determines the stability of a system response to perturbations. Robust systems return similar or identical responses when perturbed while non-robust systems return very different responses [17,18]. Biofilm antibiotic tolerance is a product of complex cellular systems. The presented study examines the robustness of colony biofilm antibiotic tolerance to industrially and medically relevant perturbations including 1) nutrient environment 2) temperature 3) quorum sensing ability and 4) growth phase.

To our knowledge, this is the first time robustness analysis has been applied to biofilm antibiotic tolerance. Antibiotic tolerance is at the heart of many practical challenges related to unwanted biofilms. Being able to predict biofilm antibiotic tolerance as a function of culturing perturbations is essential for rationally designing and evaluating antimicrobial strategies. The presented results shed insight on the varying success rates of common anti-fouling strategies like antibiotic impregnated coatings and provide a template for improved antimicrobial testing schemes.

## Results

### 1. Antibiotic tolerance in planktonic and biofilm cultures

Biofilms often exhibit very different antibiotic tolerances than planktonic cultures [1-4]. To interpret the presented biofilm data in an appropriate context, the antibiotic tolerances of biofilm cultures were compared to planktonic cultures. Antibiotics representing the aminoglycoside and beta-lactam classes were used as proxies for the diverse array of utilized agents.

**Figure 1:**
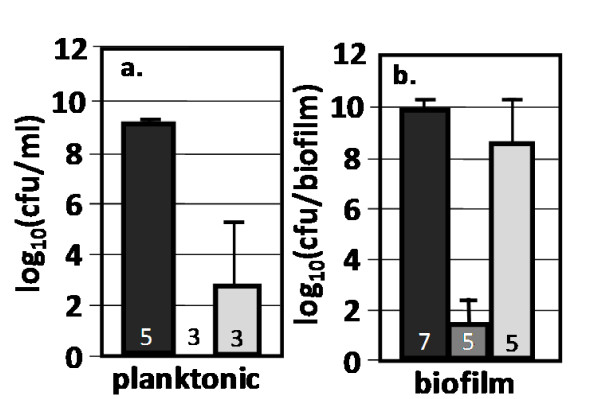
**Comparison of planktonic and biofilm antibiotic tolerance**. Wild-type *E. coli *K-12 cultures were grown on LB only medium at 37°C. Cultures were grown for 6 hours before being transferred to fresh antibiotic treatment medium for 24 hours. Reported cfu/ml and cfu/biofilm data was determined after treatment. Black bars = control, dark gray bars = kanamycin (100 ug/ml), light gray bars = ampicillin (100 ug/ml) challenge. Number at the base of each bar denotes the number of independent replicates. cfu = colony forming unit.

Kanamycin and ampicillin tolerances were determined for planktonic and biofilm cultures grown in Luria-Bertani (LB) medium at 37°C. These antibiotics were highly effective against planktonic cultures reducing colony forming units (cfu's)/ml by 6 to 9 orders of magnitude (Fig. 1a). The biofilm antibiotic tolerance results were varied. Kanamycin produced a 9 log_10 _reduction in cfu's per biofilm while ampicillin resulted in only a one log_10 _reduction in cfu's per biofilm (Fig 1b). Subsequent biofilm responses to culturing perturbations were compared to these base tolerance results (Fig. 1b). Just prior to antibiotic challenge, the biofilm cultures contained 9.3 log_10 _± 0.1 cfu's/biofilm while the planktonic cultures had 7.8 ± 0.2 log_10 _cfu's/ml. Additional data illustrating differences in colony biofilm antibiotic susceptibility as compared to planktonic cultures can be found in Additional file 1, Figs. S1 and S5.

The results reinforce the concept that biofilm cultures can behave very differently from planktonic cultures and trends from planktonic cultures may not be relevant to biofilm cultures. Considering the well established importance of biofilms in medical infections, it is essential to test antimicrobial strategies against relevant microbe growth conditions.

### 2. Nutritional perturbations

Surfaces susceptible to microbial colonization are often subjected to changing nutrient levels. For instance, a central venous catheter would experience different blood glucose levels based on patient activity, diel feeding schedules, or medical conditions like diabetes. Industrial food preparation surfaces could experience different nutrient loads based on worker schedules. The effect of nutritional environment perturbations on biofilm antibiotic tolerance was assayed to determine if antibiotic efficacy would be predictable.

**Figure 2:**
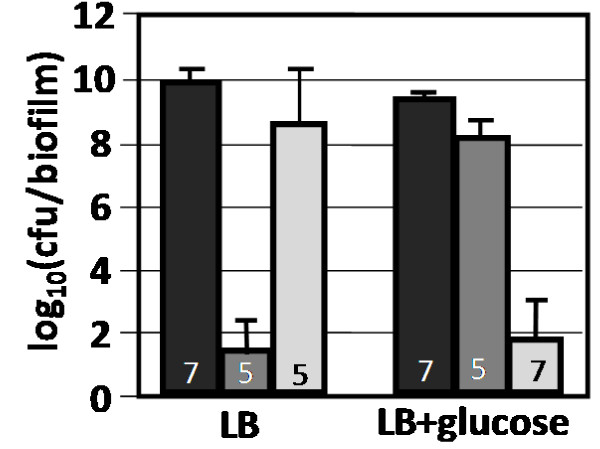
**Effect of glucose perturbation on wild-type *E. coli *K-12 biofilm antibiotic tolerance**. Cultures were grown as biofilms for 6 hours before being transferred to antibiotic treatment plates for 24 hours. Conditions included only LB medium and LB medium supplemented with 10 g/L of glucose. Reported cfu/biofilm data was determined after treatment. Black bars = control, dark gray bars = kanamycin (100 ug/ml), light gray bars = ampicillin (100 ug/ml) challenge. Number at the base of each bar denotes the number of independent replicates. cfu = colony forming unit.

Perturbing the nutritional environment by adding 10 g/L glucose to LB medium produced a large change in colony biofilm kanamycin and ampicillin tolerance (Fig. 2). In the presence of glucose, kanamycin reduced cfu's per biofilm by approximately one order of magnitude. This is in stark contrast with the 9 log_10 _decrease observed in the absence of glucose. In the presence of glucose, ampicillin produced a 7 log_10 _decrease in cfu's per biofilm. For comparison, ampicillin produced a one order of magnitude reduction in cfu's per biofilm when grown on LB only. Just prior to antibiotic challenge, the biofilm cultures grown on LB + glucose contained 8.9 ± 0.1 log_10 _cfu's/biofilm while the LB only cultures contained 9.3 ± 0.1 cfu's/biofilm. Changes in antibiotic tolerance were not likely due to different cell densities as reported with planktonic *S. aureus *cultures [19]. Interestingly, perturbing planktonic cultures with 10 g/L glucose had no statistically significant effect on kanamycin and ampicillin tolerance (Additional file 1, Fig. S1). The planktonic culture densities just prior to antibiotic challenge were 7.5 ± 0.4 log_10 _and 7.8 ± 0.2 log_10 _cfu/ml for the LB + glucose and LB only cultures respectively.

**Figure 3:**
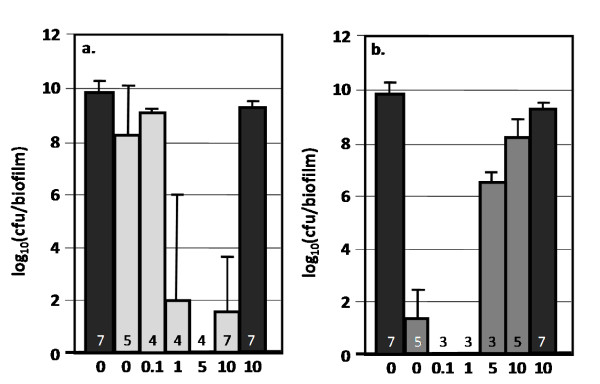
**Effect of glucose concentration on antibiotic tolerance of wild-type *E. coli *K-12 biofilm cultures**. Cultures were grown as biofilms for 6 hours before being transferred to antibiotic treatment plates for 24 hours. LB medium was supplemented with varying amounts of glucose indicated below each bar ranging from 0-10 g/L. Reported cfu/biofilm data was determined after treatment. Black bars = control, light gray bars = ampicillin (100 ug/ml) challenge. Number at the base of each bar denotes the number of independent replicates. cfu = colony forming unit.

The glucose effect was analyzed to determine what magnitude of perturbation was required to elicit the observed antibiotic tolerance changes. Five glucose concentrations were tested (Fig. 3). The biofilm cultures showed an increased sensitivity to ampicillin when the initial glucose concentration was at least 1 g/L. The shift in kanamycin tolerance was observed between initial glucose concentrations of 1 and 5 g/L. It should be noted that LB media contains trace concentrations of sugar but the quantities are not significant enough to support measurable growth in respiration negative *E. coli *[20].

**Figure 4:**
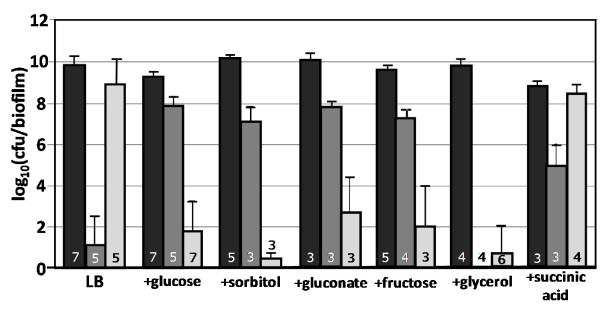
**Effect of nutritional environment on antibiotic tolerance of wild-type *E. coli *biofilm cultures**. Cells were grown as biofilms for 6 hours before being transferred to treatment plates for 24 hours. All cultures were grown at 37°C in LB medium with or without an additional carbon source. All carbon source supplements were added at 10 g/L, the succinic acid solution was pH adjusted to 6.8 before being added to medium. Reported cfu/biofilm data was determined after treatment. Black bars = control, dark gray bars = kanamycin (100 ug/ml) challenge, light gray bars = ampicillin (100 ug/ml) challenge. Number at the base of each bar denotes the number of independent replicates. cfu = colony forming unit.

The effect of glucose on antibiotic tolerance was expanded to test other common hexoses found in the human diet including the glucose isomer fructose, the more reduced sorbitol, and the more oxidized gluconate. All tested hexoses had effects analogous to glucose and made the biofilm cultures more susceptible to ampicillin (Fig. 4). Experiments also examined media augmented with the carbohydrate glycerol or the organic acid succinic acid. The presence of glycerol produced an ampicillin tolerance response similar to the hexose grown cultures and a kanamycin response similar to the LB only cultures. Cultures grown on succinic acid supplemented medium had antibiotic tolerances analogous to the LB only cultures.

**Figure 5:**
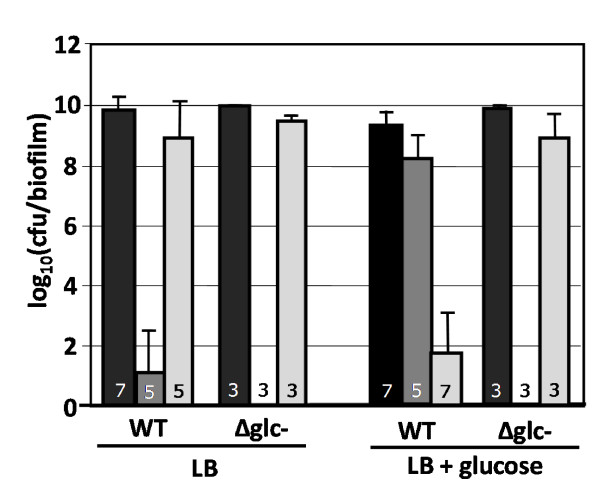
**Effect of glucose perturbation on *E. coli *K-12 biofilm culture antibiotic tolerance for wild-type and glucose negative mutants**. Cultures were grown as biofilms for 6 hours before being transferred to antibiotic treatment plates for 24 hours. Conditions included only LB medium and LB medium supplemented with 10 g/L of glucose. Reported cfu/biofilm data was determined after treatment. Δ*glc- *glucose negative *E. coli *K-12 strain (Δ*ptsG*, Δ*ptsM*, Δ*glk*, Δ*gcd*). Black bars = control, dark gray bars = kanamycin (100 ug/ml), light gray bars = ampicillin (100 ug/ml) challenge. Number at the base of each bar denotes the number of independent replicates. cfu = colony forming unit.

*E. coli *strains unable to utilize glucose were constructed by sequential deletion of the *ptsG*, *ptsM*, *glk*, and *gcd *genes using the KEIO gene knock-out library and P1 transduction methods (see materials and methods). The glucose negative cultures did not respond to glucose perturbations; antibiotic tolerance did not change significantly between the presence and absence of glucose (Fig. 5). The glucose effect appeared to be a result of metabolic adjustments, not membrane effects or the presence of an inhibitory compound.

The biofilm cultures demonstrated a non-robust antibiotic tolerance response when the nutritional environment was perturbed with carbohydrates. The data suggests that appropriate nutrient concentration ranges must be considered when evaluating antimicrobial strategies.

### 3. Temperature perturbations

Surfaces susceptible to biofilm formation are often subjected to temperature changes or gradients. For instance, a central venous catheter would experience core body temperature at the tip and room temperature at the bung. A continuous gradient would exist between these two extremes. This section's goal was to determine if the efficacy of an antibiotic would be predictable when the system temperature was perturbed.

Biofilm antibiotic tolerance was tested at temperatures above and below the human core temperature of 37°C, both in the presence and absence of glucose. The temperature range was selected to consider room temperature (21°C) relevant to many food items, industrial settings, and the external surfaces of implanted medical devices like catheters. The temperature of 42°C was selected to represent the elevated temperatures associated with pyrexia.

**Figure 6:**
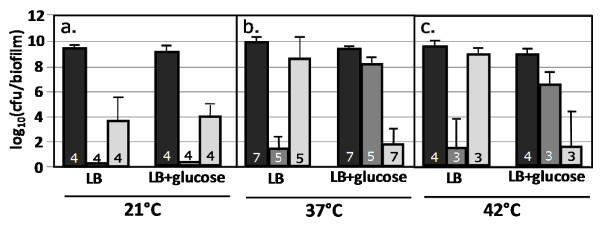
***E. coli *biofilm antibiotic tolerance as a function of temperature (21, 37, 42°C)**. Cells were grown as biofilms for 6 hours before being transferred to treatment plates for 24 hours. Reported cfu/biofilm data was determined after treatment. a) Cultures grown at 21°C, b) cultures grown at 37°C, and c) cultures grown at 42°C. Black bars = control, dark gray bars = kanamycin (100 ug/ml) challenge, light gray bars = ampicillin (100 ug/ml) challenge. Number at the base of each bar denotes the number of independent replicates. cfu = colony forming unit.

Antibiotic tolerance changed with some temperature perturbations. At 21°C, kanamycin and ampicillin reduced cfu's/biofilm by 6 to 9 orders of magnitude (Fig. 6a). This response was not affected by the presence of glucose. At 42°C, biofilm antibiotic tolerance was analogous to the results from 37°C; the cultures demonstrated a large change in kanamycin and ampicillin tolerance as a function of nutritional environment (Fig. 6b, c).

The biofilm antibiotic tolerance response is not robust to perturbations in temperature. Changes in antibiotic tolerance are not necessarily predictable *a priori*. In addition to considering nutrient environment, this data suggests it is critical to know if an antibiotic treatment will be effective over a device's operational temperature range.

### 4. AI-2 quorum sensing perturbations

Bacteria can communicate with other organisms and can sense properties related to their surroundings using small soluble molecules in a process termed quorum sensing (QS). QS has been associated with the multicellular coordination of many microbial behaviors including pathogenicity and biofilm formation (reviewed in *e.g. *[21,22]). Combining QS interference strategies with antibiotic treatments has been effective against certain microbes under certain conditions and has generated considerable scientific interest (*e.g.*[23], reviewed in [24]). The efficacy of such combined treatments under perturbed culturing conditions therefore represents a critical assessment of the general applicability of the strategy.

A set of *E. coli *AI-2 QS gene deletion mutants was constructed to act as proxies for QS interference strategies targeting different aspects of AI-2 QS. The strains lacked key enzymes in AI-2 synthesis (Δ*luxS*), phosphorylation (Δ*lsrK*), regulation (Δ*lsrR*), and degradation pathways (Δ*lsrF*) (reviewed in [25]). The AI-2 system was chosen because of its wide distribution among both Gram negative and positive organisms and because it has been shown to modulate biofilm formation [25].

The *E. coli *K-12 MG1655 AI-2 QS mutants were constructed using the KEIO gene knock-out library and P1 transduction methods (see materials and methods). The strains were characterized for planktonic and biofilm growth characteristics. Mutant and wild-type planktonic growth rates were nearly identical (Additional file1, Fig. S2). Colony biofilm growth rates and final cell densities also showed no statistical difference (Additional file1, Fig. S3). The AI-2 production profiles for planktonic cultures can be found in Additional file 1, Fig. S4. The AI-2 profiles were similar to previous reports [26-28].

**Figure 7:**
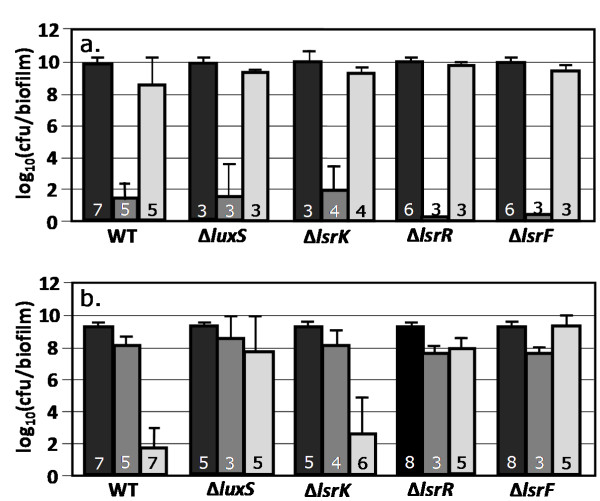
**Effect of AI-2 quorum sensing circuit gene deletions on antibiotic tolerance of *E. coli *biofilm cultures**. Cells were grown as biofilms for 6 hours before being transferred to treatment plates for 24 hours. Reported cfu/biofilm data was determined after treatment. 7a) Cultures grown at 37°C on LB only medium. 7b) Cultures grown at 37°C on LB and 10 g/L glucose. Δ*luxS *mutant lacked gene for AI-2 synthesis, Δ*lsrK *mutant lacked gene for AI-2 phosphorylation, Δ*lsrR *mutant lacked gene for lsr operon repression, and Δ*lsrF *mutant lacked gene for AI-2 degradation. Black bars = control, dark gray bars = kanamycin (100 ug/ml) challenge, light gray bars = ampicillin (100 ug/ml) challenge. Number at the base of each bar denotes the number of independent replicates. cfu = colony forming unit.

Perturbation of AI-2 QS did not result in any significant changes in biofilm antibiotic tolerance when cultured at 37°C on LB only medium (Fig. 7a). When the AI-2 QS deletion mutants were perturbed with glucose, non-intuitive changes in antibiotic tolerance were observed. Deleting genes associated with AI-2 synthesis (Δ*luxS*), regulation (Δ*lsrR*), or degradation (Δ*lsrf*) increased ampicillin antibiotic tolerance. These cultures had 6 orders of magnitude more cfu's/biofilm after ampicillin treatment as compared to both wild-type and AI-2 phosphorylation (Δ*lsrK*) mutants. Additional experimental data regarding the effects of AI-2 gene deletions on antibiotic tolerance can be found in Additional file 1, Figs. S5-S9. Interestingly, the Δ*luxS *mutant demonstrated an altered glucose catabolite repression response.

The results suggest *E. coli *biofilm antibiotic tolerance is robust to perturbations in AI-2 QS when grown on LB at 37°C however; the response becomes non-robust in the presence of glucose. The results indicate that QS interference can have unpredictable results that change as a function of targeted gene and culturing perturbations.

### 5. Colony biofilm antibiotic tolerance and culture stage

The data presented in Figs. 1, 2, 3, 4, 5, 6 and 7 were collected from biofilm cultures grown for 6 hours prior to the 24 hour antibiotic challenge. At 6 hours, the biofilm cultures were still growing (Additional file 1, Fig. S3). Additional experiments examined antibiotic tolerance when the biofilm cultures were grown for 12 or 24 hours prior to antibiotic challenge. At these time intervals, the cultures would be in early and established stationary phase (Fig. S3).

**Figure 8:**
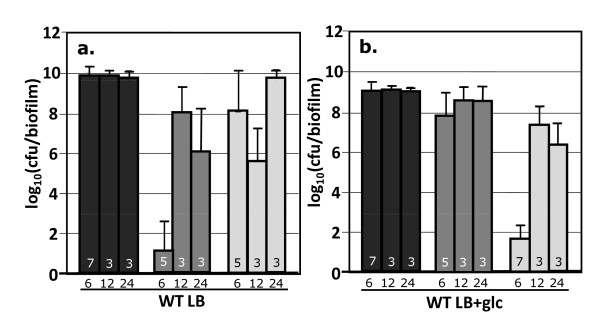
**Effect of culturing phase on antibiotic tolerance of wild-type *E. coli *K-12 cultures**. Cells were grown as biofilms for 6, 12, or 24 hours prior to being transferred to treatment plates. Cultures treated after 6 hours were in late exponential phase while the 12 and 24 hour samples were in stationary phase. Reported cfu/biofilm data was determined after treatment. Cultures were grown at 37°C. 8a) LB only medium. 8b) LB and 10 g/L glucose. Black bars = control, dark gray bars = kanamycin (100 ug/ml) challenge, light gray bars = ampicillin (100 ug/ml) challenge. Number at the base of each bar denotes the number of independent replicates. cfu = colony forming unit.

When grown on LB only, there was a growth stage dependent change in antibiotic tolerance. For instance, cultures grown for 12 hours prior to ampicillin challenge had 7 orders of magnitude more culturable cells per biofilm than cultures grown for 6 hours prior to challenge (Fig. 8a). When cultures were grown on LB + glucose, no significant, culturing phase dependent kanamycin tolerance effect was observed (Fig. 8b). The biofilm cultures grown in the presence of glucose did show a culturing stage dependent tolerance to ampicillin. A 6 log_10 _difference in cfu's per biofilm was observed between the samples grown for 6 and 12 hours prior to antibiotic challenge.

Colony biofilms exhibited a non-robust antibiotic tolerance as a function of growth stage. This was anticipated. Antibiotics are generally more effective against dividing cells than stationary phase cells. Therefore, the lack of a growth stage dependent kanamycin tolerance in the presence of glucose was surprising. Depending on the specific antibiotic and the specific culturing condition, the effect of growth stage on antibiotic tolerance may not be predictable. The results once again highlight the necessity of appropriate growth conditions when testing anti-biofilm strategies.

## Discussion

The current study examined the robustness of colony biofilm antibiotic tolerance as a function of culturing perturbations. *E. coli *antibiotic tolerance was not robust. Perturbations in nutritional environment, temperature, AI-2 QS ability, and biofilm age resulted in very different, context specific, responses. Relatively small perturbations like increasing the initial glucose concentration from 0.1 to 1 g/L, resulted in a 7 log_10 _difference in culturable cells per biofilm after ampicillin challenge. Human blood glucose levels average approximately 1 g/L. Changes in blood glucose levels due to diel cycles, fasting, or diabetes could significantly change a biofilm's susceptibility to antibiotic treatments. A summary of the tolerance responses can be found in Table 1. To facilitate cross experiment comparisons, the log reduction (LR) in cfu's/biofilm between control and challenged cultures was determined. The difference between the smallest LR and the largest LR for a set of culturing conditions was determined for 1) LB +glucose vs. LB only, 2) culturing at 37°C vs. 21 and 42°C, 3) wild-type cultures vs. AI-2 QS deletion mutants as well as for the aggregate perturbations 4) glucose and temperature and 5) glucose and AI-2 QS mutants. The only perturbation to elicit a robust response for both kanamycin and ampicillin was AI-2 QS interference. However, this response was not robust when multiple perturbations were considered. Aggregate perturbations always resulted in a larger ΔLR indicating a less robust response. Taken together, the data in Table 1 demonstrate that antibiotic tolerance is highly susceptible to perturbations.

**Table 1:** Summary of *E. coli *K-12 biofilm antibiotic tolerance robustness analyses

	kanamycin			ampicillin		
**perturbation**	**low LR^1^**	**high LR^1^**	**ΔLR^2^**	**low LR^1^**	**high LR^1^**	**ΔLR^2^**

glucose	1.3	8.8	7.5	1.5	7.6	6.1

temperature	8.4	9.5	1.1	0.5	5.8	5.3

AI-2 QS	8.8	9.9	1.1	0.3	1.5	1.2

culture stage	1.7	8.8	7.1	0.1	4.6	4.5

glucose + temp.	1.3	9.5	8.2	0.5	7.6	7.1

glucose+AI-2 QS	0.8	9.9	9.1	0.3	7.6	7.3

1. For each set of perturbation data, the lowest and highest log reduction (LR) in cfu's/biofilm are listed. The perturbed conditions are compared to biofilm cultures grown on LB only medium at 37°C. cfu = colony forming unit.

**2. **ΔLR = the maximum observed range in log reductions (LR) between the base scenario

and the perturbed culturing condition.

This study examined antibiotic tolerance in the model organism *E. coli*. While this organism was selected for its extensive literature base and its convenient molecular biology systems, some *E. coli *strains are serious pathogens. For instance, there are uropathogenic strains associated with recurrent bladder and kidney infections, adherent-invasive strains associated with Crohn's disease [29], and diarrhoeagenic strains which are responsible for an estimated 2 × 10^5 ^to 2 × 10^6 ^deaths per year [30]. The lack of a robust antimicrobial tolerance response observed with this model organism is likely relevant to a wide range of enterobacter as well as other microorganisms. This study examined the no shear colony biofilm system. Other biofilm culturing systems which apply different levels of shear or use different substratum may influence antibiotic susceptibility as suggested in [31].

Antibiotic tolerance is a complex emergent property of numerous cellular systems. The observed changes in antibiotic tolerance are likely the result of numerous cellular mechanisms. Nutritional environment had a large effect on observed antibiotic tolerance. The role of carbon source and anaerobiosis on antibiotic tolerance has been reported for decades using planktonic cultures (*e.g. *[32,33]) and more recently using biofilm cultures [34]. The proposed mechanisms are varied and could involve complex changes in many cellular systems including membrane structure, alterations of transmembrane potential, and the expression of different genes including multidrug efflux pumps [35-39]. Many of these cellular properties have been reported to change as a function of biofilm associated genes including ycfR(bhsA) or as a function of growth phase based indole secretion [40-42].

Based on the changes in antibiotic tolerance as a function of glucose, the current data suggests the cAMP-catabolite repression protein (cAMP-CRP) circuit may play a role in antibiotic tolerance. Intracellular cAMP levels are widely reported to change in the presence of sugars [43,44]. These effects are often associated with the PTS sugar transporter systems. Glycerol and gluconate are not imported via the PTS family of transporters but both influence the *E. coli *cAMP-CRP catabolite repression system through undetermined mechanisms [45,46]. Interestingly, augmenting LB with glycerol made the wild-type cultures highly sensitive to both kanamycin and ampicillin. This was not observed with any other supplemented carbon source hinting at some unknown aspect of glycerol metabolism. Adding both glycerol (10 g/L) and glucose (10 g/L) to the LB resulted in antibiotic tolerance trends analogous to the LB + glucose medium, consistent with anticipated glucose repression effects (data not shown). This would indicate that increased antibiotic sensitivity in LB + glycerol was not directly due to glycerol permeabilization of the cellular membrane but rather a metabolic effect.

The cultures grown at 21°C were generally more susceptible to both kanamycin and ampicillin. At this temperature, there was no observed difference in antibiotic tolerance as a function of glucose perturbations. The absence of a nutritional effect suggests the cAMP-CRP regulatory system is influenced by temperature. Additional cellular processes could also be contributing to the observed behaviors including temperature dependent changes in multidrug pump expression [40], temperature dependent changes in cellular membrane properties [47] and temperature dependent changes in growth rate. A biofilm grown at 21°C for 6 hours would be less established than a biofilm grown at 37°C for 6 hours. While Fig. 8 shows a growth stage dependent change in ampicillin tolerance, it does not show a growth stage dependent change in kanamycin tolerance when glucose is present. The changes in antibiotic tolerance at 21°C were for both kanamycin and ampicillin suggesting it is not just a growth stage dependent phenomenon.

Interrupting AI-2 QS had varied and unpredictable effects on antibiotic tolerance. A growing body of research suggests different organisms use QS for different purposes and that QS effects can be quite diverse. For instance, a recent review highlights that the *luxS *based AI-2 QS system can increase, decrease, or have no effect on biofilm formation depending on the organism or strain [25]. While acylhomoserine lactone (AI-1) based QS interference has been generally successful with *Pseudomonas aeruginosa *[23,48], accessory gene regulator (Agr) based QS interference with *Staphylococcus aureus *and *Staphylococcus epidermidis *can make the microbes more resilient to antibiotic treatments (reviewed in [49]). The current study demonstrates a large increase in antibiotic tolerance when the AI-2 QS system was disrupted however, this effect was gene and context dependent (Fig. 7). For unknown reasons, the Δ*lsrK *strain behaved analogous to the wild-type culture when perturbed with glucose. The Δ*lux*S strain was further characterized and found not to display a glucose dependent antibiotic tolerance response (Additional file1) implying a disruption of a portion of the glucose repression circuit. The Δ*lux*S strain did display catabolite repression based diauxic growth. The strain was grown on defined M9 medium containing both glucose and xylose. Like the wild-type strain, the Δ*lux*S strain preferentially consumed glucose (data not shown). The data from this study do not support pursuing a strategy of AI-2 quorum sensing interference as an antifouling approach with *E. coli*.

## Conclusions

Robustness analysis revealed that colony biofilm antibiotic tolerance is very sensitive to culturing perturbations. These tolerance responses can vary based on single or aggregate perturbations and are, in many cases, not predictable. The collective data represents both challenges and opportunities for the rational design of anti-biofilm strategies. The data demonstrates that biofilms can be countered effectively with some antibiotics if the appropriate conditions are applied however, if inappropriate conditions are applied, the efficacy of the treatment can be negated. The results indicate it is essential to evaluate antimicrobial strategies over a range of perturbations relevant to the targeted application so that accurate predictions regarding efficacy can be made.

## Methods

### Bacterial strains and growth conditions

*E. coli *K-12 MG1655 gene deletion mutants were constructed using the KEIO mutant library and P1 transduction techniques [50,51]. *E. coli *cultures were grown in low salt Luria-Bertani (LB) broth with or without different substrate supplements. When added, the supplements were autoclaved separately from the LB medium. The average starting pH of the medium was 6.8. All antibiotics were utilized at a final concentration of 100 ug/ml. The tested antibiotics had different molecular weights so this mass concentration represents a different molar concentration for each agent. Culturing temperatures ranged from 21 to 42°C depending on experiment.

### Colony biofilm culture antibiotic tolerance testing

The colony biofilm culturing method has been described previously [3,4,7,52,53]. Briefly, colony biofilm systems consist of agar plates, sterile 0.22 μm pore- 25 mm diameter polycarbonate membranes (GE Water and Process Technologies, K02BP02500), and the desired bacterial strains. The membrane is placed aseptically on agar plates and inoculated with 100 uL of an exponentially growing culture (diluted to OD_600 _= 0.1). The culture is grown for 6 hours on untreated plates of the desired medium composition. After the initial growth phase, the biofilm is aseptically transferred to either a treated or a control plate where it is incubated for an additional 24 hours. The nutrients and antibiotics enter the biofilm from below the membrane. Antibiotic penetration of colony biofilms has been studied expensively suggesting the agent readily moves throughout the biofilm [3]. The delivery of antibiotic is diffusion based analogous to the many antibiotic impregnated coating systems. After treatment, the colony biofilms are aseptically transferred to 10 ml glass test tubes pre-filled with 5 mL of sterile phosphate buffered saline. The colony biofilm is vortexed vigorously for 1 minute to separate the cells from the membrane. The membrane is removed and discarded. The dislodged biofilm is homogenized using a tissue homogenizer for 40 seconds to ensure complete physical disaggregation. The homogenized culture is serially diluted and colony forming units (cfu's) per membrane are enumerated using the drop-plate method [54].

### Planktonic culture antibiotic tolerance testing

For planktonic antibiotic tolerance experiments, 50 ml cultures were grown exponentially for six hours with shaking (250 ml flask, 150 rpm) at 37°C in untreated medium (with or without 10 g/L glucose). The cells were collected using centrifugation (800 rcf, 20 minutes). The cells were resuspended in fresh medium of noted composition and cultured for another 24 hours at 37°C with shaking (150 rpm). The viable cell counts were determined using serial dilutions and the drop-plate cell enumeration method [54]. All cultures were grown in the presence of atmospheric oxygen.

### Deletion mutant generation

*E. coli *K-12 MG1655 gene deletion mutants were constructed using the KEIO knock-out library, P1 transduction methods, and wild-type *E. coli *strain MG1655 [50,51]. The strains were verified using PCR and physiological studies.

### Statistical analysis of results

Statistical significance was determined using p-values from unpaired T-tests of experimental and control samples. All error bars represent standard error of 3 to 8 replicates.

## Abbreviations

cfu: colony forming unit; QS: quorum sensing; LR: log reduction

## Authors' contributions

Conception and design of experiment: TRZ, RPC. Acquisition of data: TRZ, HB, JLR, LJT. Analysis and interpretation of data: TRZ, PSS, RPC. Drafting the manuscript: PSS, RPC. Revising the manuscript critically for intellectual content: TRZ, HB, PSS, RPC. Final approval of published version: TRZ, HB, JLR, LJT, PSS, RPC.

## Supplementary Material

Additional file 1 Supplementary culture data. This file contains supporting planktonic and biofilm culture. Click here for file
