# Supplementary material for: Robustness analysis of culturing perturbations on Escherichia coli colony biofilm beta-lactam and aminoglycoside antibiotic tolerance
Source: BMC Microbiol. 2010 Jul 7;10:185. doi: 10.1186/1471-2180-10-185 (PMC2912858; doi:10.1186/1471-2180-10-185)
Supplement: Additional file 1 — Supplementary culture data. This file contains supporting planktonic and biofilm culture. [file 1471-2180-10-185-S1.PDF]

**Supplemental file material:**

# Robustness analysis of biofilm antibiotic tolerance

Trevor R. Zuroff, Hans Bernstein, Jennifer Lloyd-Randolfi, Lourdes Jimenez Taracido, Philip S. Stewart, Ross P. Carlson

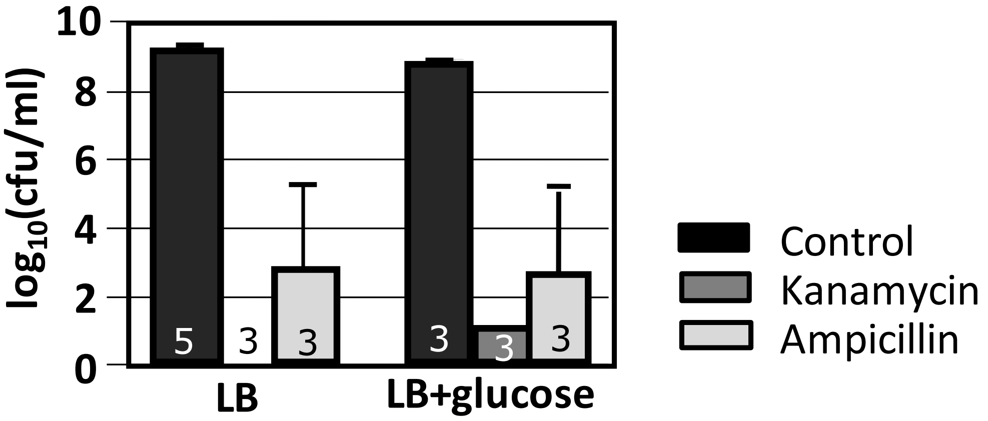


**Figure S1. Planktonic culture antibiotic tolerance as a function of nutritional environment perturbations.** Cells were grown exponentially in planktonic culture for 6 hours before being transferred to treatment medium for 24 hours. Reported cfu/ml data was determined after treatment. Cultures were grown at 37°C on LB only or LB supplemented with 10g/L glucose. Black bars = control, dark gray bars =kanamycin (100ug/ml) challenge, light gray bars = ampicillin (100ug/ml) challenge. Number at the base of each bar denotes the number of independent replicates. cfu = colony forming unit.

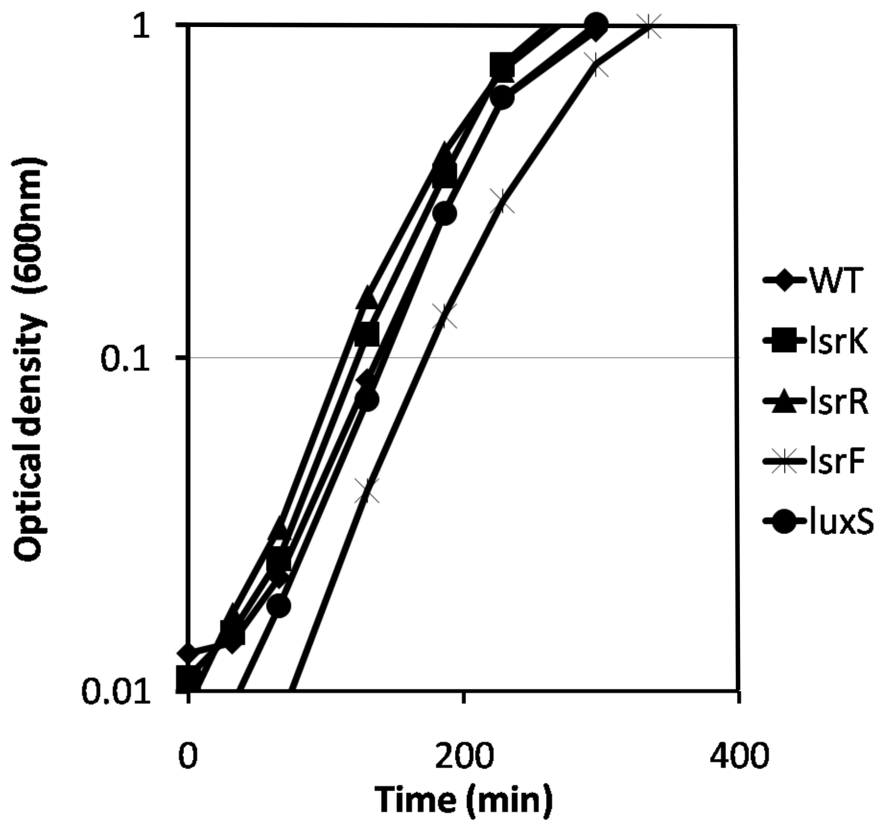


**Figure S2. Planktonic growth curves for wild-type *E. coli* K-12 and four AI-2 quorum sensing gene knock out mutants (∆*lsrK,* ∆*lsrR,* ∆*lsrF,* ∆*luxS*).** Culture density is expressed as optical density (600 nm). Note the y-axis is a log scale so the specific growth rate is proportional to the slope of the optical density versus time line. The specific growth rates were very similar regardless of the AI-2 quorum sensing circuit gene knock-out. Average specific growth rate 1.2/hr. Cultures grown in 250 ml shake flasks on 50 ml of LB + 1% glucose at 33°C and 150 rpm.

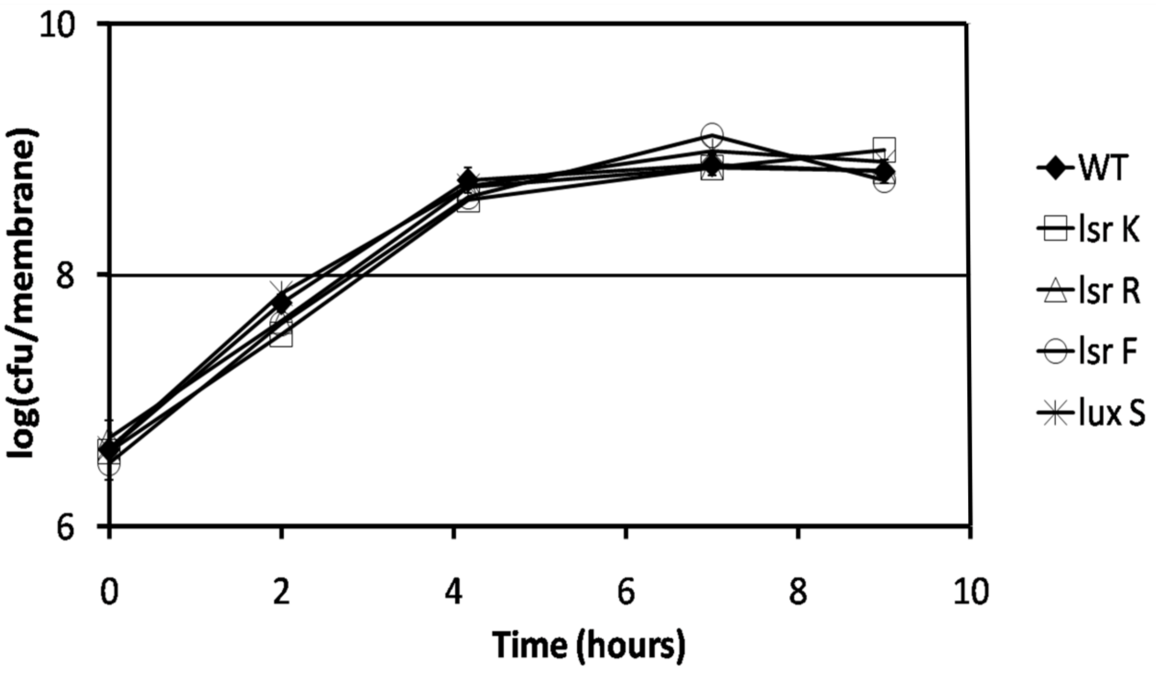


**Figure S3. Comparison of colony biofilm growth rates for wild-type *E. coli* K-12 and four AI-2 quorum sensing knock out mutants (Δ*lsrK,* Δ*lsrR,* Δ*lsrF,* Δ*luxS*).** Specific growth rates and final cell densities were very similar regardless of the AI-2 quorum sensing circuit gene knock-out. See main text for experimental details. Cultures grown on LB + 1% glucose agar plates at 37°C.

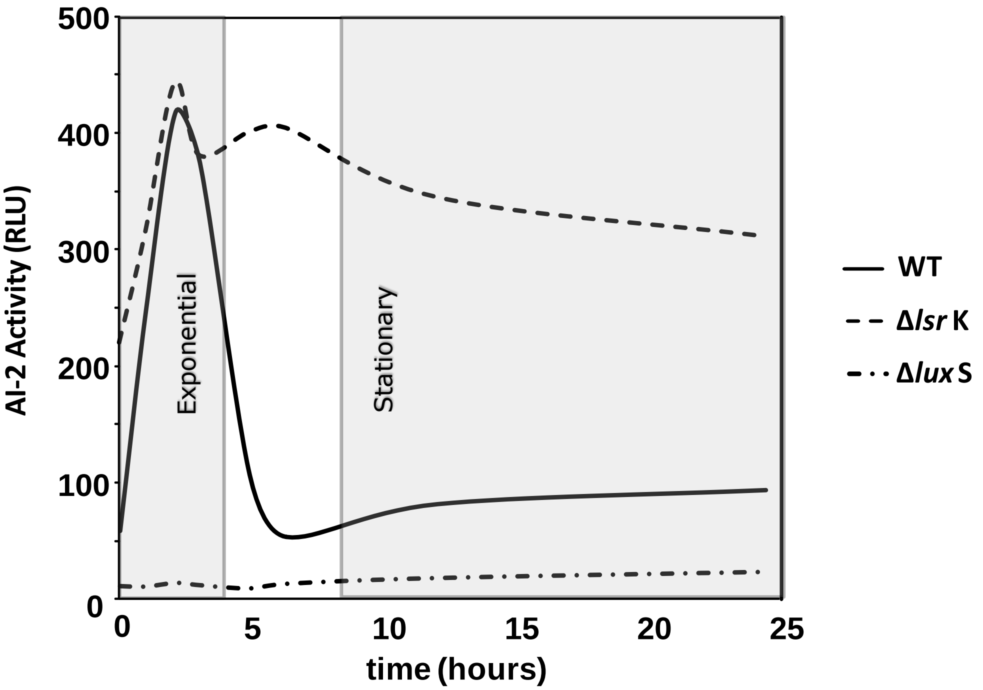


**Figure S4. AI-2 accumulation as a function of batch planktonic growth for wild-type *E. coli* MG1655 and AI-2 mutants Δ*lsrK* and Δ*luxS*.** AI-2 levels are reported based on the *Vibrio harveyi* bioluminescence assay and plotted as relative light units (RLU). Shaded areas highlight exponential and stationary phase of batch growth. The area in between shaded regions represents the transition from exponential growth to stationary phase. Data is representative from three separate experiments. Cultures grown in 250 ml shake flasks on 50 ml of LB medium at 37°C and 150 rpm.

***Vibrio harveyi* AI-2 activity assay.** *E. coli* culture supernatants were tested for the presence of AI-2 by measuring the induction of luminescence in V. *harveyi* reporter strain MM32 (kindly provided by Dr. B. Bassler). Cultures of MM32, innoculated from freezer stocks, were grown overnight at 150 rpm and 30°C in AB medium (17.5 g/L NaCl, 12.3 g/L $MgSO_4$, 2 g/L casamino acids adjusted to pH 7.5 with KOH. After sterilization, 10 mL/L of 1M $KH_2PO_4$, 10mL/L of 0.1 M L-arginine, and 20 mL/L of 50% (v/v) glycerol were added, modified from Greenberg et al., 1979). After overnight growth, the cultures were diluted 1:1000 in fresh AB medium and placed on ice. Frozen *E. coli* cell-free culture supernantents were thawed on ice. Diluted MM32 and *E. coli* supernatants were added to the wells of black 96 well microtiter plates (10µL *E. coli* supernatant to 90µL MM32 culture). The microtiter plates were incubated at 30°C and 150 rpm. Measurements were taken approximately every two hours using a Bio-Tek FL600 Microplate Fluorescence Reader. Luminosity is recorded as relative light units (RLU) and cell density is measured as $OD_{600}$. Reported luminosity values were normalized with respect to *V. harveyi* culture $OD_{600}$. All reported comparisons were made

from samples analyzed from the same microtiter plate with aliquots from the same MM32 culture.

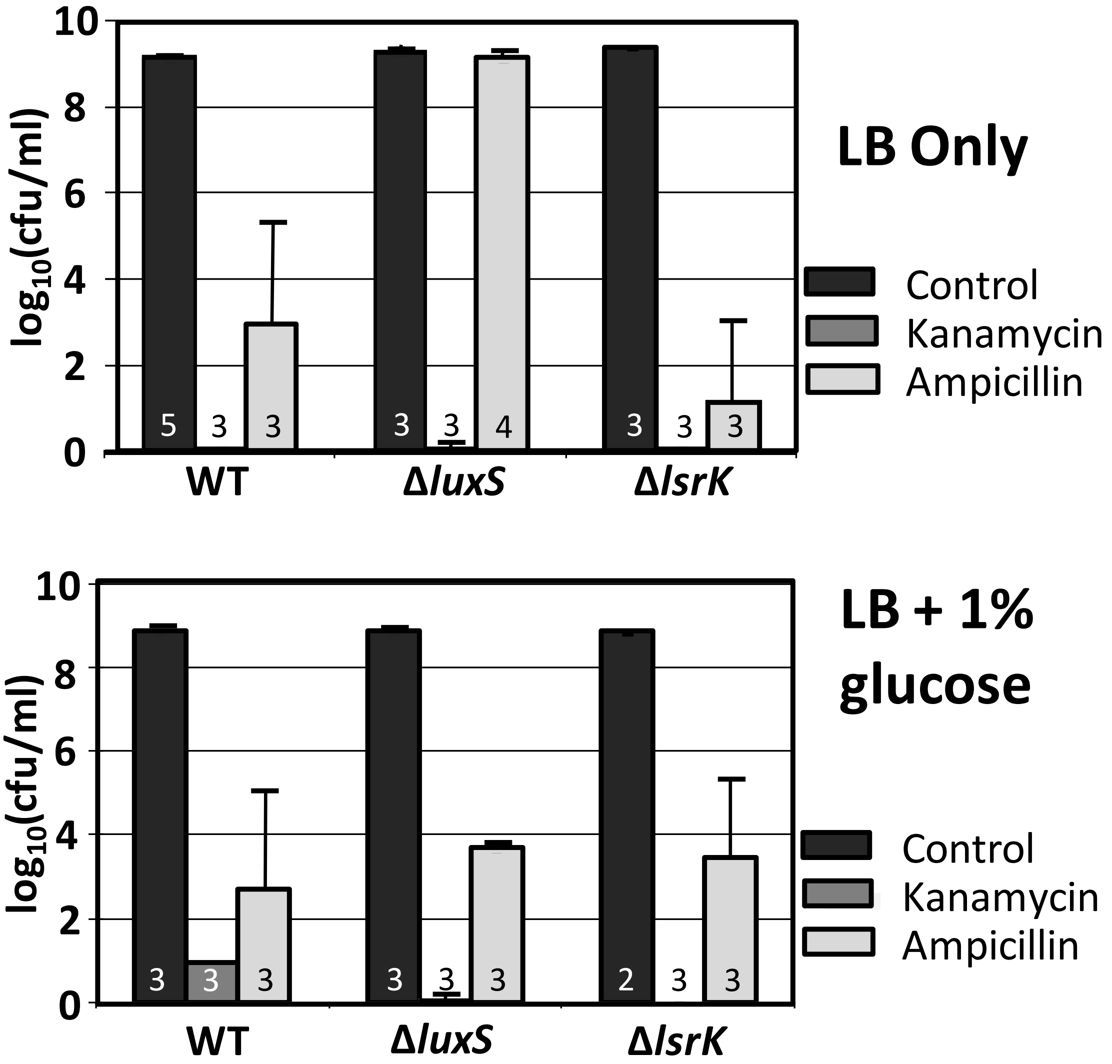


**Figure S5. Effect of AI-2 quorum sensing circuit gene knock-outs on antibiotic tolerance of planktonic *E. coli* cultures.** Cells were grown exponentially in planktonic culture for 6 hours before being transferred to treatment medium for 24 hours. Reported cfu/ ml data was determined after treatment. A) Cultures grown at 37°C on LB only medium. B) Cultures grown at 37°C on LB and 10g/L glucose. Black bars = control, dark gray bars =kanamycin (100ug/ml) challenge, light gray bars = ampicillin (100ug/ml) challenge.  Number at the base of each bar denotes the number of independent replicates. cfu = colony forming unit.

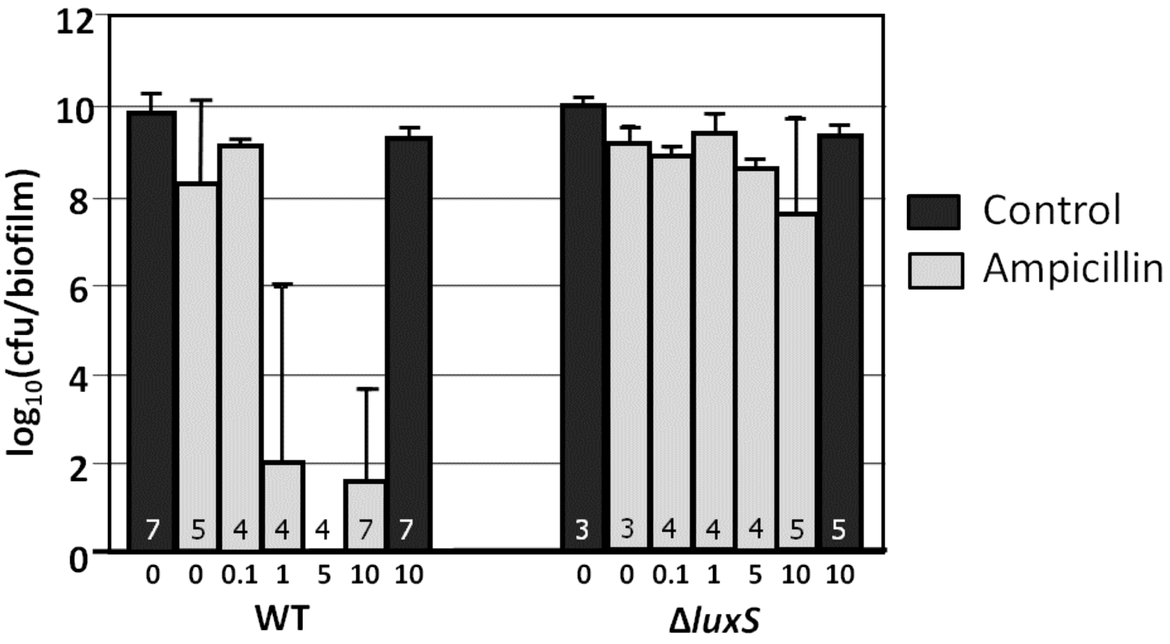


**Figure S6. Effect of glucose concentration on antibiotic tolerance of wild-type and *luxS* gene knock-out mutant *E. coli* biofilm cultures.** Cells were grown as biofilms for 6 hours before being transferred to treatment plates for 24 hours. LB medium was supplemented with varying amounts of glucose indicated below each bar ranging from 0-10 g/L. Reported cfu/ biofilm data was determined after treatment. Black bars = control, light gray bars = ampicillin (100ug/ml) challenge. Number at the base of each bar denotes the number of independent replicates. cfu = colony forming unit.

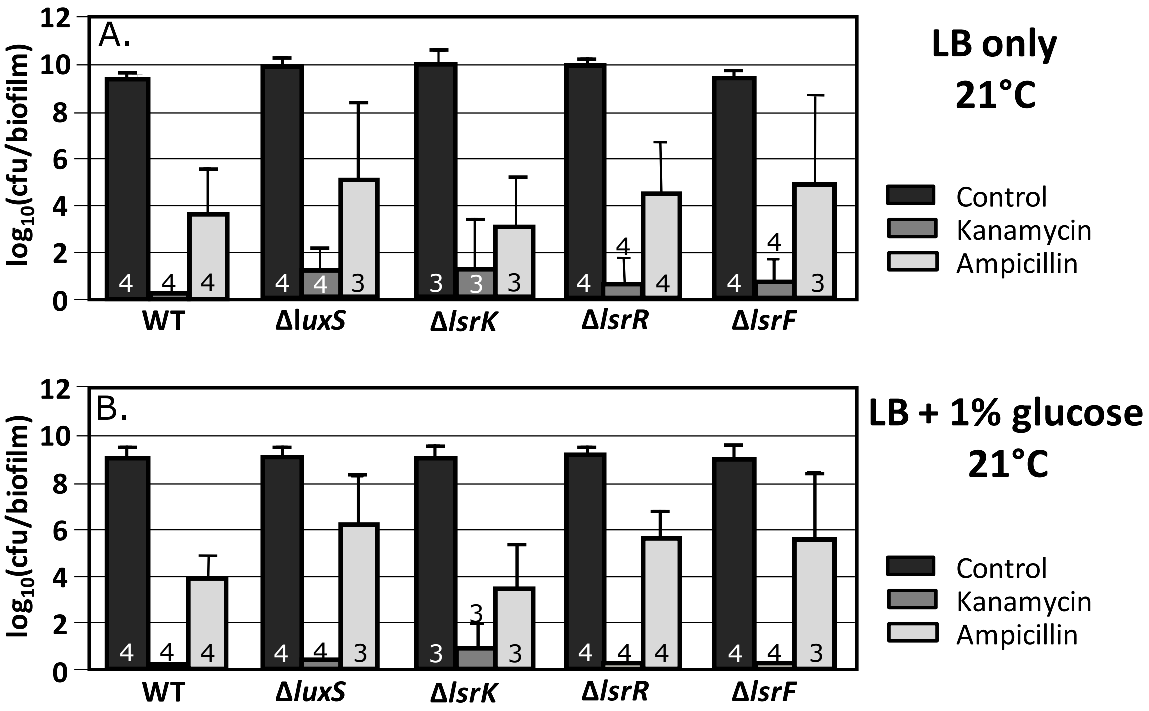


**Figure S7. *E. coli* biofilm antibiotic tolerance as a function of AI-2 quorum sensing circuit gene knock-outs, nutritional environment, and temperature (21°C).** Cells were grown as biofilms for 6 hours before being transferred to treatment plates for 24 hours. Reported cfu/ biofilm data was determined after treatment. A) Cultures grown at 21°C on LB only medium. B) Cultures grown at 21°C on LB and 10g/L glucose. Black bars = control, dark gray bars =kanamycin (100ug/ml) challenge, light gray bars = ampicillin (100ug/ml) challenge.  Number at the base of each bar denotes the number of independent replicates. cfu = colony forming unit.

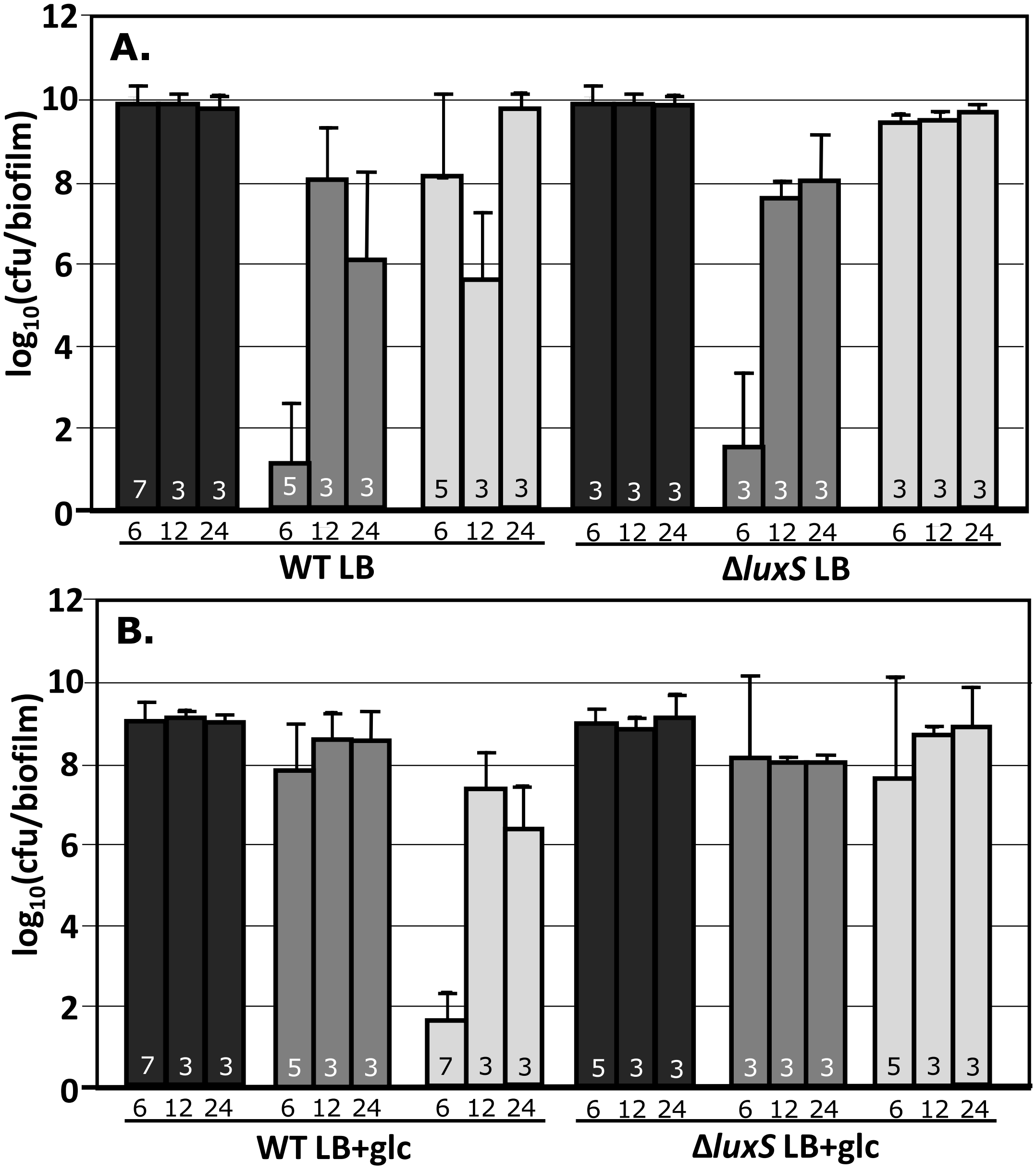


**Figure S8. Effect of culturing phase on antibiotic tolerance of wild-type and Δ*luxS* biofilm cultures.** Cells were grown as biofilms for 6, 12, or 24 hours before being transferred to treatment plates for 24 hours. Cultures treated after 6 hours were in late exponential phase while the 12 and 24 hour samples were in stationary phase. Reported cfu/ biofilm data was determined after treatment. Cultures were grown at 37°C. A) LB only medium. B) LB and 10g/L glucose. Black bars = control, dark gray bars =kanamycin (100ug/ml) challenge, light gray bars = ampicillin (100ug/ml) challenge. Number at the base of each bar denotes the number of independent replicates. cfu = colony forming unit.

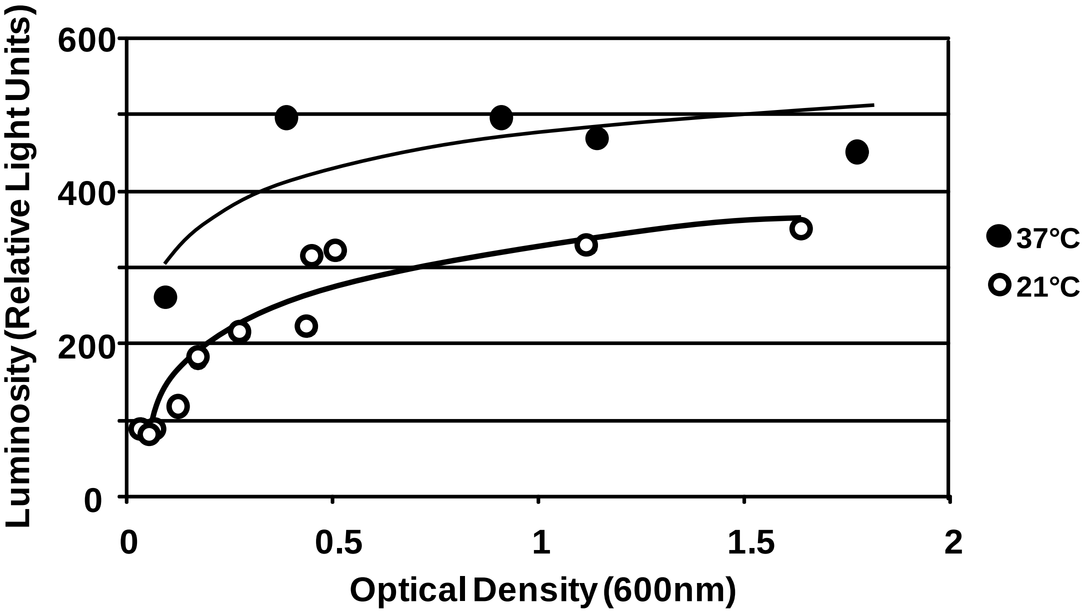


**Figure S9. AI-2 levels as a function of temperature for planktonic, wild type *E. coli* MG1655 cultures.** AI-2 levels expressed as relative light units based on the *Vibrio harveyi* bioluminescence assay are plotted as a function of culture optical density (OD 600nm). Filled circle data collected from cells grown at 37°C and open circle data collected from culture grown at 21°C. Data is representative of three separate experiments.
